# Association between monocyte-to-high-density lipoprotein-cholesterol ratio and gallstones in U.S. adults: findings from the National Health and Nutrition Examination Survey 2017–2020

**DOI:** 10.1186/s12944-024-02166-1

**Published:** 2024-06-07

**Authors:** Xingwu Liu, Guanyu Yan, Boyang Xu, Mingjun Sun

**Affiliations:** 1 Department of Gastroenterology, The First Hospital of China Medical University, Shenyang, China; 2 Department of Endoscopy, The First Hospital of China Medical University, Shenyang, China

**Keywords:** Monocyte-to-high-density lipoprotein-cholesterol ratio, Gallstone, NHANES, Cross-sectional study

## Abstract

**Background:**

Studies have indicated that monocyte-to-high-density lipoprotein cholesterol ratio (MHR) can be a reliable indicator of various diseases. However, the association between MHR and gallstone prevalence remains unclear. Therefore, this study aimed to explore any potential association between MHR and gallstone prevalence.

**Methods:**

This study used data from the National Health and Nutrition Examination Survey (NHANES) 2017–March 2020. MHR was calculated as the monocyte count ratio to high-density lipoprotein cholesterol levels. Multiple logistic regression models, Cochran-Armitage trend test, and subgroup analyses were used to examine the association between MHR and gallstones.

**Results:**

This study included 5907 participants, of whom 636 (10.77%) were gallstone formers. The study participants had a mean age of 50.78 ± 17.33 years. After accounting for multiple covariables, the multiple logistic regression model showed a positive linear association between MHR and gallstone odds. The subgroup analyses and interaction testing results revealed that the association between MHR and gallstones was statistically different across strata, including sex, smoking, asthma, and hypertension.

**Conclusions:**

Gallstone prevalence positively associated with elevated MHR, indicating that MHR can be employed as a clinical indicator to assess gallstone prevalence.

## Introduction

Gallstone is one of the most prevalent digestive system diseases, with a global prevalence of approximately 10% [1, 2]. Its prevalence has doubled in the U.S. in the last three decades and has become a serious health concern [3]. Gallstones occur in various forms and can be divided into three categories based on their makeup: cholesterol, bile pigment, and mixed stones, with cholesterol stones accounting for over 60% of gallstones. According to their location, gallstones can be divided into gallbladder and bile duct stones [4]. The main symptoms of gallstones include abdominal pain, nausea, and vomiting. However, serious and fatal complications such as gallbladder perforation, pancreatitis, and intestinal obstruction may occur in some patients [5–7]. Gallstones are one of the main risk factors for gallbladder cancer, and are also linked to a higher risk of gallbladder cancer mortality [8, 9]. Gallstones may also increase the risk of proximal colon cancer [10]. Furthermore, over 800,000 cholecystectomy procedures are performed due to gallstones each year, costing over $6 billion in the United States [11]. Therefore, increased attention should be paid to gallstones, and identifying reliable clinical indicators of gallstone prevalence is crucial.

Previous research shows that oxidative stress and inflammation are significant factors in gallstone development. Inflammation is linked to crystal and gallstone formation in bile [12]. Inflammatory cytokines can alter the absorptive and secretory functions of human gallbladder epithelial cells, leading to gallstone formation [13]. Inflammation can also alter lipid metabolism, lead to changes in the metabolism of cholesterol and bile acids, and increase bile salt levels [14]. Patients with gallstones experience high oxidative stress levels in the gallbladder mucosa, which can lead to alterations in the gallbladder’s absorption and secretory functions, thereby promoting gallstone formation [15]. Gallstone formation is closely associated with metabolic syndrome [16], and among the components of metabolic syndrome, obesity, dyslipidemia, and insulin resistance can influence gallstone formation [17–19]. Despite these potential etiologic and risk factors for gallstones, there are insufficient clinical indicators for assessing its prevalence.

The monocyte-to-high-density lipoprotein cholesterol ratio (MHR) is a newly developed composite metric utilized as a possible clinical indicator of atherosclerosis, coronary heart disease, and other diseases because of its simple accessibility and clinical relevance [20, 21]. High-density lipoprotein cholesterol (HDL-C) acts as an anti-inflammatory factor by inhibiting macrophage migration [22]. Monocytes are a type of inflammatory cell that can migrate to the site of inflammation and accelerate oxidative stress [23]. MHR, based on the anti-inflammatory qualities of HDL-C and the pro-inflammatory qualities of monocytes, can indicate inflammation and oxidative stress, which are the potential pathogenesis of gallstones. In addition, MHR has been associated with insulin resistance, which is a risk factor for gallstones [24–26]. However, the association between MHR and gallstones remains unclear. Therefore, an in-depth exploration of the association between MHR and gallstones will provide valuable insights for further research.

To our knowledge, no population-based study has examined the association between MHR levels and gallstone formation. Therefore, we conducted this study utilizing data from the National Health and Nutrition Examination Survey (NHANES) 2017–March 2020 to ascertain the association between MHR levels and gallstones.

## Materials and methods

### Study population

The NHANES research program aimed to evaluate the nutritional status and overall health of people in the United States. Each year, the survey covers approximately 5,000 people in a nationally representative sample. The NHANES comprises five items of data: demographic, dietary, examination, laboratory, and questionnaire data. The general public has free access to all these data.

**Fig. 1:**
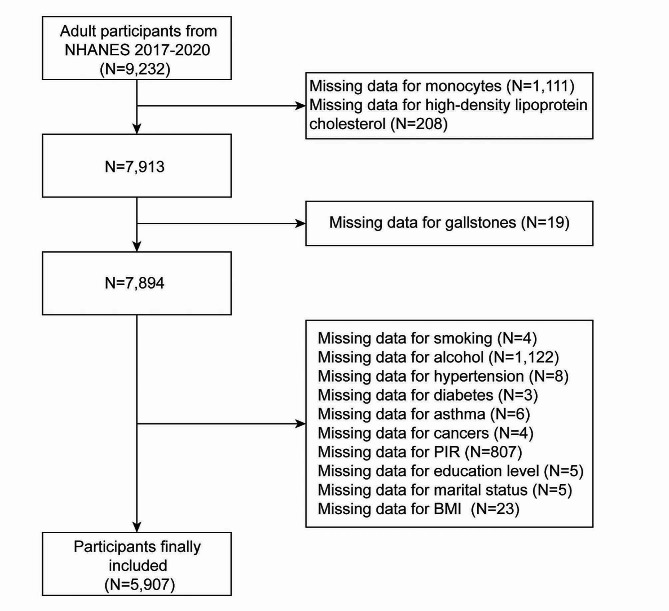
Flowchart of participants selection. *Abbreviations*: NHANES, National Health and Nutrition Examination Survey; PIR, Income to poverty ratio; BMI, Body mass index

Owing to the availability of gallstone information in the questionnaire data, this study only used data from the NHANES 2017–March 2020 to explore the association between MHR and gallstones. Initially, 15,560 people were included, of which 9,232 aged ≥ 20 years were retained. However, 1319 people who lacked information on MHR and 19 who lacked information on gallstones were excluded. Furthermore, 1987 people who lacked information about covariables including education level, marital status, income-to-poverty ratio (PIR), body mass index (BMI), alcohol consumption, smoking, asthma, cancer, hypertension, and diabetes were excluded. Ultimately, 5907 people were included and Fig. 1 displayed the details of selection criteria of the study population.

### Assessment of MHR and gallstone

MHR was designed as an exposure variable calculated as the ratio of monocyte counts (10^3^ cells/uL) to HDL-C level (mmol/L). The complete blood count of the blood specimens was measured using a Beckman Coulter DxH 800 instrument, and each participant was provided with a distribution of blood cells. An endpoint reaction specific to HDL-C was used to measure HDL-C levels. The reaction product was measured photometrically at 600 nm.

Gallstones were used as outcome variables. The questionnaire “Has a doctor or other health professional ever told you that you had gallstones?” was used to assess the presence of gallstones.

### Covariables

A multivariable adjustment model was constructed to summarize potential confounding factors and determine whether they affected the association between MHR and gallstones. First, demographic data, including sex, age, race, education level, marital status, and PIR were included. Second, the examination data—BMI—was included. Third, questionnaire data on smoking, alcohol consumption, hypertension, diabetes, cancer, and asthma were included. Alcohol consumption was defined as “the consumption of any type of alcoholic beverage at least once in a month during the past 12 months.” Smoking was defined as having consumed more than 100 cigarettes in the past. Medical comorbidities such as diabetes, hypertension, cancer, and asthma were ascertained through self-reported history in the questionnaires.

### Statistical analysis

Sample weights were used to calculate estimates using the combined 2017 to March 2020 data to ensure the reliability of this dataset in generating national estimates. Depending on whether gallstones were present or absent, participants were divided into two groups. A log2 transformation was used for MHR because of its skewed distribution, and then it was analyzed as a continuous variable. For continuous variables, the mean ± standard deviation was used to report the distribution of variables, whereas frequencies and percentages were reported for categorical variables to provide a thorough perspective. To evaluate the differences based on the distribution and categorization of the variables and data, the Student’s t-test or chi-squared test was employed. A multiple logistic regression model was used to examine the association between MHR and gallstones in the three distinct models. Model 1 remained unadjusted for covariables. Demographic data was adjusted in Model 2 and all covariables were adjusted in Model 3. In addition, a Cochran-Armitage trend test was conducted to analyze the dose-response of MHR to gallstone prevalence. MHR was categorized into tertiles, with tertile 1 as the reference group. Subgroup analyses and interaction tests were performed. Stratification was based on covariables to examine differences in the above associations.

R software 4.3.2 and EmpowerStats 4.2 were used for statistical analyses. Statistical significance was set at *P* < 0.05.

## Results

### Baseline characteristics

Following these selection criteria, this study included 5907 individuals, 49.38% females and 50.62% males. Study participants’ baseline characteristics were illustrated in Table 1. Depending on whether gallstones were present or absent, participants were divided into two groups, with 636 stone formers and 5271 non-stone formers. Compared with non-stone formers, gallstone formers tend to be older, female, non-Hispanic white, obese (BMI ≥ 30), smokers, and have hypertension, cancers, asthma, and diabetes (*P* < 0.05). However, non-stone formers were more likely to be drinkers and never married compared with stone formers (*P* < 0.05).

**Table 1:** Baselines characteristics of participants

Characteristic	Total (*N* = 5,907)	Non-stone formers	Stone formers	*P*-value
		(*N* = 5,271)	(*N* = 636)	
**Age (years)**	50.78 ± 17.33	49.93 ± 17.33	57.89 ± 15.57	< 0.001
**Gender (%)**				< 0.001
Male	2990 (50.62%)	2805 (53.22%)	185 (29.09%)	
Female	2917 (49.38%)	2466 (46.78%)	451 (70.91%)	
**Race (%)**				< 0.001
Mexican American	694 (11.75%)	611 (11.59%)	83 (13.05%)	
Other Hispanic	575 (9.73%)	505 (9.58%)	70 (11.01%)	
Non-Hispanic White	2315 (39.19%)	2017 (38.27%)	298 (46.86%)	
Non-Hispanic Black	1483 (25.11%)	1369 (25.97%)	114 (17.92%)	
Other Race	840 (14.22%)	769 (14.59%)	71 (11.16%)	
**Education level (%)**				0.315
Less than high school	930 (15.74%)	820 (15.56%)	110 (17.30%)	
High school	1429 (24.19%)	1268 (24.06%)	161 (25.31%)	
More than high school	3548 (60.06%)	3183 (60.39%)	365 (57.39%)	
**Marital Status (%)**				< 0.001
Married/Living with Partner	3459 (58.56%)	3070 (58.24%)	389 (61.16%)	
Widowed/Divorced/Separated	1350 (22.85%)	1175 (22.29%)	175 (27.52%)	
Never married	1098 (18.59%)	1026 (19.46%)	72 (11.32%)	
**Income to poverty ratio (%)**				0.093
≤ 1	1079 (18.27%)	973 (18.46%)	106 (16.67%)	
> 1-<4	3159 (53.48%)	2793 (52.99%)	366 (57.55%)	
≥ 4	1669 (28.25%)	1505 (28.55%)	164 (25.79%)	
**BMI (kg/m** ^**2**^ **) (%)**				< 0.001
< 25	1429 (24.19%)	1363 (25.83%)	66 (10.46%)	
> 25-<30	1849 (31.30%)	1675 (31.75%)	174 (27.58%)	
≥ 30	2629 (44.51%)	2238 (42.42%)	391 (61.96%)	
**Alcohol (%)**				< 0.001
Yes	3049 (51.62%)	2801 (53.14%)	248 (38.99%)	
No	2858 (48.38%)	2470 (46.86%)	388 (61.01%)	
**Smoked (%)**				0.004
Yes	2698 (45.67%)	2373 (45.02%)	325 (51.10%)	
No	3209 (54.33%)	2898 (54.98%)	311 (48.90%)	
**Asthma (%)**				< 0.001
Yes	962 (16.29%)	829 (15.73%)	133 (20.91%)	
No	4945 (83.71%)	4442 (84.27%)	503 (79.09%)	
**Cancers (%)**				< 0.001
Yes	647 (10.95%)	532 (10.09%)	115 (18.08%)	
No	5260 (89.05%)	4739 (89.91%)	521 (81.92%)	
**Hypertension (%)**				< 0.001
Yes	2274 (38.50%)	1935 (36.71%)	339 (53.30%)	
No	3633 (61.50%)	3336 (63.29%)	297 (46.70%)	
**Diabetes (%)**				< 0.001
Yes	1071 (18.13%)	888 (16.85%)	183 (28.77%)	
No	4836 (81.87%)	4383 (83.15%)	453 (71.23%)	
**MHR**	0.46 ± 0.24	0.46 ± 0.24	0.47 ± 0.23	0.067

### The association between MHR and gallstones

The findings of this study indicated that gallstones were more prevalent among individuals with a higher MHR (Table 2). This association was not significant in Model 1 (unadjusted model); however, it was statistically significant in Model 2 and Model 3 (odds ratio (OR) = 1.27; 95% confidence interval (CI): 1.11–1.46), indicating that the odds of gallstones increased by 27% for every unit rise in log2-transformed MHR. To further explore whether the association between MHR and gallstones was dose-dependent, a Cochran-Armitage trend test was performed by transforming the continuous variable MHR into a categorical variable (tertiles), with tertile 1 as the reference group. The test results showed that tertiles 2 and 3 had higher gallstone odds than did the lowest tertile group (tertile 1), which was statistically significant in Models 2 and 3, indicating a significant linear association between MHR and gallstones.

**Table 2:** The associations between MHR and the odds of gallstone

Characteristic	Model 1 OR (95%CI)	Model 2 OR (95%CI)	Model 3 OR (95%CI)
Log2-transformed MHR	1.22 (1.00, 1.27)	1.43 (1.25, 1.63)	1.27 (1.11, 1.46)
MHR Tertiles			
Tertile 1	Reference	Reference	Reference
Tertile 2	1.13 (0.92, 1.39)	1.37 (1.11, 1.70)	1.25 (1.00, 1.55)
Tertile 3	1.21 (0.99, 1.49)	1.74 (1.39, 2.17)	1.46 (1.16, 1.83)
*P* for trend	0.0686	< 0.0001	0.0014

### Subgroup analysis and interaction testing

Subgroup analyses were conducted to determine whether the association between MHR and gallstones were robust. The results in Table 3 indicated an inconsistent association between MHR and gallstones. The results of the interaction testing revealed that the association between MHR and gallstones was statistically different across strata, including sex, smoking, asthma, and hypertension (*p* for interaction < 0.05). The positive association between MHR and gallstones was significant for participants who were female, non-smokers, had asthma or did not have hypertension. Conversely, the association was not statistically significant for participants who were male, smokers, without asthma, or with hypertension.

**Table 3:** Subgroup analysis between MHR and the prevalence of gallstone

Subgroup characteristic	OR (95%CI)	*P* for interaction
**Gender**		0.0196
Male	0.93 (0.55, 1.56)	
Female	2.13 (1.29, 3.52)	
**Smoked**		0.0346
Yes	1.03 (0.65, 1.65)	
No	2.16 (1.30, 3.59)	
**Asthma**		0.0424
Yes	2.86 (1.37, 5.99)	
No	1.20 (0.83, 1.73)	
**Hypertension**		0.0411
Yes	1.11 (0.72, 1.69)	
No	2.32 (1.31, 4.10)	

## Discussion

Following strict selection criteria, 5907 participants were included in this study. According to this study, MHR is positively related to gallstone prevalence, with a 27% increase in gallstone odds per unit increase in log2-transformed MHR in a fully adjusted model. A trend test demonstrated a dose-response for this association. Subgroup analysis and interaction testing revealed that the association between MHR and gallstones was statistically different across strata, including sex, smoking, asthma, and hypertension. Based on these results, it can be hypothesized that MHR is a potential clinical indicator of gallstone prevalence.

Notably, several studies utilizing the NHANES database have identified MHR as an indicator of various diseases. For example, a study that included over 30,000 participants demonstrated that MHR was substantially associated with cardiovascular mortality in the American population [27]. Another study investigated the association between MHR and coronary heart disease and found a positive association between MHR and the prevalence of coronary heart disease at an MHR < 0.6 [28]. Moreover, numerous studies have demonstrated that MHR is not only linked to cardiovascular disease, but may also be associated with several other diseases. Zhou et al. found MHR was negatively associated with the risk of osteoporosis in females [29]. According to Wang et al., higher MHR was linked to a higher prevalence of nonalcoholic fatty liver disease (NAFLD) among Americans [30]. However, no study has examined the association between MHR and gallstones. Therefore, we investigated the possible association between MHR and gallstones using data from the NHANES database. The multiple logistic regression model showed a positive linear association between MHR and gallstone odds. This result is beneficial for identifying asymptomatic gallstone patients in clinical practice. The diagnosis of asymptomatic gallstones requires imaging examinations such as abdominal ultrasound. While MHR was calculated as the ratio of monocyte counts to HDL-C level. Given that whole blood cell counts and analysis of blood lipid profiles are routine examinations, the availability of MHR is much higher compared to abdominal ultrasound data. Therefore, MHR can be a novel and convenient indicator for the prevalence of gallstones, and can help clinical doctors decide whether patients should undergo abdominal ultrasound examination in the future. In addition, the subgroup analyses and interaction testing results revealed that the association between MHR and gallstones was statistically different across strata, including sex, smoking, asthma, and hypertension. In females, higher MHR was associated with a higher gallstone prevalence. This may be because of higher estrogen levels in females, which promotes cholesterol blockage and influences gallstone formation [31]. This finding may be attributed to obesity in patients with asthma. Obesity is a well-recognized complication of asthma that alters the metabolism of the liver and gallbladder, among other metabolic organs. These alterations include increased bile secretion, hyperlipidemia, and reduced intestinal peristalsis, which in turn affects the development of gallstones [32, 33]. Nicotine, the main component of cigarettes, has an anti-estrogenic effect that reduces the saturation of cholesterol in bile, speeds up gallbladder emptying, and affects the development of gallstones [34]. As for hypertension, it may be attributed to leptin, a hormone secreted by fat cells. Leptin elevates blood pressure and affects biliary motility. Patients with hyperleptinemia are more likely to develop gallstones [35–37]. The mechanism of these findings is unclear, further prospective studies are needed to better understand the association between MHR and gallstone prevalence across subgroups.

At present, the potential mechanisms behind this positive association between MHR and gallstones prevalence are not well elucidated. Multiple mechanisms might be involved. First, during inflammation, circulating monocytes are derived from macrophages, which become the major population in inflammation and are crucial in the inflammatory and oxidative responses to gallbladder stones [38, 39]. Second, monocytes mediate the overabundance of reactive oxygen species, a crucial component in gallstone pathogenesis [40]. Third, dyslipidemia is strongly associated with gallstones. A previous study has shown that decreased HDL-C levels increase the risk of gallstones in older patients with NAFLD [41]. A recent study demonstrated a negative linear association between serum HDL-C levels and the risk of developing gallstones in European populations [42]. The results of these studies provide solid and reliable evidence to explain the findings of this study, which states that elevated MHR levels are positively associated with increased gallstone prevalence.

## Strengths and limitations

This study has several strengths. First, the data in the NHANES database were subjected to rigorous quality control and were nationally representative. Second, confounding covariables were adjusted to reduce the effect of bias on the findings. Third, to determine whether there were any population-specific differences in the association between MHR and gallstone prevalence, subgroup analyses were performed. There are, however, some limitations to this study. First, this study only included data from 2017 to March 2020 because of the availability of gallstone data. Second, despite considering and including multiple covariables, we could not ascertain that the effects of all potential confounders had been excluded. Third, the diagnosis of gallstones was based on questionnaires and lacked a more precise imaging diagnosis. In addition, data from NHANES were based on self-reported questionnaires, which meant that there was an inevitable recall bias. Ultimately, this was a cross-sectional study, and the causal association between MHR and gallstones could not be clarified.

## Conclusion

In conclusion, the present study found that a higher MHR was linked to a higher prevalence of gallstones, suggesting that MHR can be used as a clinical indicator to assess gallstone prevalence. This could help personalize interventions and assist physicians in determining which groups of people can be screened for gallstones to reduce the economic burden and their serious complications.

## Data Availability

This study is based on the public database, and all related-datasets are available at https://www.cdc.gov/nchs/nhanes/.

## Abbreviations

MHR: Monocyte-to-high-density lipoprotein-cholesterol ratio
NHANES: National Health and Nutrition Examination Survey
HDL-C: High-density lipoprotein-cholesterol
PIR: Income to poverty ratio
BMI: Body mass index
OR: Odds ratio
CI: Confidence interval
NAFLD: Nonalcoholic fatty liver disease
